# Occurrence of Mycotoxins and Toxigenic Fungi in Cereals and Application of Yeast Volatiles for Their Biological Control

**DOI:** 10.3390/toxins14060404

**Published:** 2022-06-13

**Authors:** Asma Alkuwari, Zahoor Ul Hassan, Randa Zeidan, Roda Al-Thani, Samir Jaoua

**Affiliations:** Environmental Science Program, Department of Biological and Environmental Sciences, College of Arts and Sciences, Qatar University, Doha 2713, Qatar; asma.alkuwarii@gmail.com (A.A.); zahoor@qu.edu.qa (Z.U.H.); rzedan@qu.edu.qa (R.Z.); rathani@qu.edu.qa (R.A.-T.)

**Keywords:** toxigenic fungi, ochratoxin A, aflatoxins, wheat, rice, maize, oats, breakfast cereals, biocontrol, food safety

## Abstract

Fungal infections in cereals lead to huge economic losses in the food and agriculture industries. This study was designed to investigate the occurrence of toxigenic fungi and their mycotoxins in marketed cereals and explore the effect of the antagonistic yeast *Cyberlindnera jadinii* volatiles against key toxigenic fungal strains. *Aspergillus* spp. were the most frequent contaminating fungi in the cereals, with an isolation frequency (Fr) of 100% in maize, followed by wheat (88.23%), rice (78.57%) and oats (14.28%). Morphological and molecular identification confirmed the presence of key toxigenic fungal strains in cereal samples, including *A. carbonarius*, *A. flavus*, *A. niger*, *A. ochraceus* and *A. parasiticus*. Aflatoxins (AFs) were detected in all types of tested cereal samples, with a significantly higher level in maize compared to wheat, rice, oats and breakfast cereals. Ochratoxin A (OTA) was only detected in wheat, rice and maize samples. Levels of mycotoxins in cereals were within EU permissible limits. The volatiles of *Cyberlindnera jadinii* significantly inhibited the growth of *A. parasiticus*, *A. niger* and *P. verrucosum*. The findings of this study confirm the presence of toxigenic fungi and mycotoxins in cereals within the EU permissible limits and the significant biocontrol ability of *Cyberlindnera jadinii* against these toxigenic fungi.

## 1. Introduction

Throughout the world, dietary starch and proteins are mainly obtained from cereals [1]. Other nutritional components of cereals include fiber, non-starch carbohydrates, lipids, minerals and vitamins [2]. Because of their high nutritive values, good health effects and their availability, cereals have been an essential source of human food for millions of years [3]. In the year 2022, estimated cereal production is 2799 million tons, with a high proportion of coarse grains, wheat, maize and rice [4]. However, cereal crops are prone to several biotic and abiotic stressors, among which fungal infections are considered a major biotic factor, rendering deceased and/or low quality cereals [5]. Mold contamination of the cereals occurs at different stages of their growth, processing and preservation [6]. These infections can be categorized as pathogenic, for infections leading to plant diseases and low productivity, and toxigenic, for infections resulting in the accumulation of toxic metabolites affecting productivity. In both cases, the quality and quantity of the cereals is compromised, leading to huge annual economic losses in the agricultural sector worldwide [7]. 

Fungi, particularly those from the genera *Aspergillus*, *Penicillium* and *Fusarium*, might accumulate, during target infection, secondary metabolites known as mycotoxins. Among the 300 known mycotoxins, aflatoxins (AFs), synthesized by *A. flavus* and *A. parasiticus*; ochratoxin A (OTA), synthesized by *A. carbonarius*, *A. ochraceus*, *A. westerdijkiae*, and some strains of *A. niger*; and Zearalenone (ZEN), synthesized by *F. graminearum* and *F. culmorum*, are widely studied due to their significant health effects [8]. In 2004, Abdulkadar and others [9] detected AFs in 3 out of 5 basmati rice samples in the range of 0.14–0.24 µg/kg, while the other varieties of rice were free from AF contamination. The presence of OTA, DON and ZEN were also confirmed in the rice samples in their study. Similarly, wheat and corn samples were found contaminated with *Fusarium* mycotoxins (such as DON and ZEN) only. More recently, *Aspergillus*, *Penicillium* and *Fusarium* fungi and their toxins were detected in cereals intended for animal feed in the state of Qatar [10,11]. 

A huge range of toxic effects of mycotoxins are reported in people exposed to cereals contaminated with mycotoxins. Depending on the nature of the mycotoxin and their exposure dose, the effects may be mild gastrointestinal, growth retardation, immunosuppression, teratogenicity, mutagenicity, etc. [12]. In keeping toxicity data, world food regulatory bodies have set regulatory limits for some mycotoxins. However, in recent years, there are increasing reports of the (simultaneous) occurrence of multiple mycotoxins in the food chain [13,14]. The presence of several mycotoxins in food commodities is due to either (a) the contamination of a commodity with different fungal species, (b) the production of more than one mycotoxin by a single fungus, or (c) the mixing of cereals contaminated with different mycotoxins. Although the individual mycotoxin levels are within the permissible limits, the synergistic or additive toxic effects may pose huge risks to the exposed communities [15,16,17]. This developing dilemma of the co-occurrence of fungal toxins in food and the setting of regulatory limits by the food and health regulatory authorities can be understood better through adequate mycotoxin interaction data. 

Conventionally toxigenic fungi are identified on the basis of their colony morphology, such as their size, shape, color, sporulation, etc. Such a method is not only time consuming but also requires expertise in the field of fungal identification [18]. Recently, species-specific PCR primers targeting ITS regions have been designed for the accurate and early identification of fungal species [19,20]. This molecular identification technique allows foe the differentiation of closely related fungal species, which is relatively difficult to achieve through morphological identification. Moreover, the amplification of cluster genes involved in mycotoxin synthesis pathways can be a helpful tool in differentiating potentially toxigenic fungal strains from non-toxigenic ones [10,21]. 

Although it is impossible to prevent fungal infection and mycotoxin accumulation in cereals, it is essential to minimize the levels of toxins in food. There are several chemical and physical approaches to inhibit the growth and spread of fungi in cereal crops and their products [22]. Fungicides are effective in preventing fungal growth, but the accumulation of synthetic chemicals in the food chain renders these cereals unacceptable for the consumer. Currently, consumers prefer green-labeled, minimally proceeded and high-quality products, driving the food industry towards bio-preservation, which, in fact, safeguards nutritive quality and organoleptic characteristics. Several in vitro studies reported successful application of friendly yeast and bacterial strains against toxigenic fungi [23,24,25]. The volatile organic compounds (VOCs) of *Lachancea thermotolerans*, identified mainly as 2-phenylethanol, significantly inhibited *A. parasiticus*, *P. verrucosum* and *F. graminearum* in artificial media and suppressed their potential to synthesize mycotoxins. Additionally, the VOCs of *Lachancea thermotolerans* inhibited the germination and spread of *F. oxysporum* spores inoculated on tomato leaf surfaces [23]. In another study [25], a yeast, *Kluyveromyces marxianus* QKM-4, isolated from a local dairy product (laban), inhibited several fungal species from the genera *Aspergillus*, *Penicillium* and *Fusarium*. Moreover, this strain was able to inhibit OTA synthesis by *A. carbonarius* and *P. verrucosum* by 98.7% and 99.6%, respectively. Microbial volatiles and diffusible organic compounds are being tested for their antifungal activities to replace the application of chemical pesticides on food crops. 

This study was designed to investigate the presence of toxigenic fungi and mycotoxins in cereals marketed in Qatar. A PCR-based approach using specific primers was applied due to its suitability for identifying toxigenic fungi from cereal foods. Finally, an antagonistic yeast strain was applied for the biocontrol of toxigenic fungi. The novelty of this study consists in the validation of molecular techniques for the early and reliable identification of toxigenic mycobiota in cereals and in the potential application of *Cyberlindnera jadinii* in the protection of cereals, particularly during their storage, from the toxigenic fungal growth and synthesis of mycotoxins. 

## 2. Results and Discussion

### 2.1. Fungal Contamination of Cereal Samples

Fungal contamination of cereals showed the highest contamination in maize, followed by wheat, rice and oats. All the samples of breakfast cereals were free from fungal contamination, which can be attributed to the impact of heat treatment during processing, killing the fungal spores. Another reason could also be the quality of the cereal, which may be augmented by the fact that there was no OTA detected in the breakfast cereal samples (Section 2.1). The presence of AFs in all cereal samples and the absence of fungal communities together suggest the killing of fungal spores during processing. The average contamination of the grain samples with at least one fungal colony was 7.5% (oats), 52.7% (maize), 16.1% (rice) and 23.2% (wheat). The contamination of cereal grains with mycotoxigenic fungi has been reported by several researchers [10,21,26]. In a similar, study conducted on animal feed cereals marketed in Qatar [18], there were higher percentages of grains contaminated with mycotoxigenic fungi in the animal feed samples. Relatively lower contamination was noticed in the present study, which could be associated with the good quality of the cereals for human consumption. Damaged grains, either by insects or due to other physical factors, can also lead to fungal infections and mycotoxin contamination. In the present study, all the cereals were of good quality and apparently free from any damage. 

There are several reports on the prevalence of mycotoxigenic fungi in food cereals. *Aspergilli* is the most important food spoilage fungi. In Iran [27], *A. flavus* was the major producer of AFs in marketed rice and other cereals. In the Romanian cereals, maize was the most contaminated commodity, among others (wheat and barley), with *Aspergilli*, particularly *A. flavus* and *A. fumigatus* [28]. In the present study, *Aspergillus* was the most prominent fungi in wheat, rice, maize and oats (Table 1). 

All maize samples (100%) were contaminated with *Aspergilli*, followed by wheat (88.23%), rice (78.57%) and oats (14.28%). Oats were the cereals least contaminated with fungal communities. Among all the isolated fungi, *Aspergillus* constituted 73.91% in rice, 67.65% in wheat, 65.95% in maize and 55.56% in oats. In line with the present study, in Tunisia, [29] detected the highest prevalence of *Aspergillus* spp. in maize, particularly *A. flavus* and *A. parasiticus*. 

### 2.2. Morphological Identification of the Isolated Fungi

In the present study, fungal species were initially identified on the basis of their morphological characteristics, including colony size, shape, color (observe and reverse), spore size and shape and microscopic appearance [30]. Two fungi, *A. flavus* and *A. parasiticus,* showed closely related morphological characteristics, such as greenish-yellow, olive green or deep green conidia on CYA. Similarly, the size of the colonies on CYA was 60–70 mm, and on MEA it was 50–70 mm. The differentiation between these two species was made by microscopic examination. *A. flavus* produced conidia having different sizes and shapes, with relatively thin walls, and ranging from smooth to rough. On the other hand, *A. parasiticus* conidia were spherical with thick and rough walls. Furthermore, *A. flavus* vesicles were larger, reaching 50 µm in diameter and bearing metulae, while *A. parasiticus* vesicle diameters rarely exceeded 30 µm and metulae were not seen. These findings were in line with the key characteristics described by Pitt and Hocking [30]. In accordance with the present study, *A. flavus* from four districts in Kenya was identified in maize and soil samples using the morphological approach [31]. In 2014, Iheanacho et al. [32] also used the same morphological and molecular approaches to identify and distinguish *A. flavus* and *A. parasiticus* from the compound feed samples in South Africa. Two other species, *A. niger* and *A. carbonarius,* both producing black sporulation, were differentiated on the basis of conidia color. In *A. niger*, the conidia color was brownish black, while in the case of *A. carbonarius,* it was jet black.

### 2.3. Molecular Identification of Mycotoxigenic Fungi Isolated from Cereal Samples

**Figure 1:**
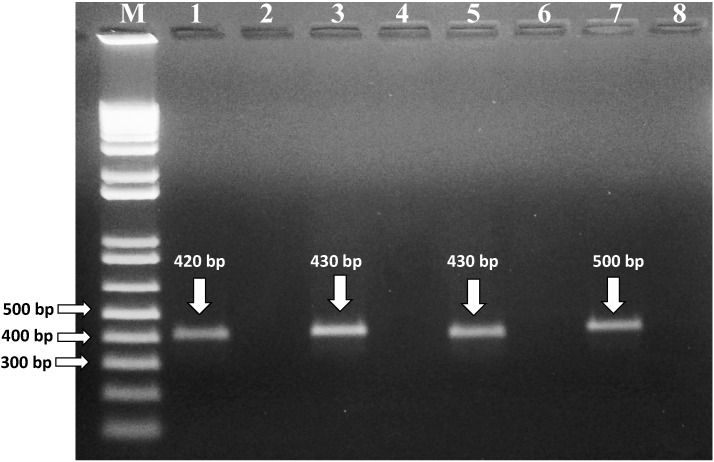
PCR amplification using species-specific primers for fungal isolates. Lanes: 1, primer ITS1/NIG with DNA from *A. niger* (amplicon size 420 bp); 3, primer PAR1/PAR2 with template DNA from *A. parasiticus* (amplicon size 430 bp); 5, primer OCRAF/OCRAR with DNA from *A. ochraceus* (amplicon size 430 bp); 7, primer FLA1/FLA2 and DNA from *A. flavus* (amplicon size 500). Lanes 2, 4, 6 and 8 represent the non-template control of their previous lane. Lane M, 1kb plus DNA marker with fragment sizes 12,000, 11,000, 10,000, 9000, 8000, 7000, 6000, 5000, 4000, 3000, 2000, 1000, 850, 650, 500, 400, 300, 200 and 100 bp.

Universal primers (ITS1/ITS4) amplified all DNA samples with a single band of 600 bp confirming that the quality of DNA was appropriate for PCR reactions [33]. Species-specific PCR primers were used for the amplification of DNA samples from the selected fungal isolates. Six isolates (coded as 3MZd, 9MRc, 9MRg) showed specific amplification using ITS1/NIG primer pair (product size 420 bp), confirming the species to be *A. niger* (Figure 1). Three isolates (3RCb, 5OTe, 4WTa) produced a single amplification band of 500 bp, using FLA1/FLA2 primer pair, confirming the species as *A. flavus*. One isolate (1WTb) was confirmed as *A. parasiticus* as it showed a single amplification band (430 bp) with the primer pair PAR1/PAR2. Two isolates (4MZd, 5 MZa) showed an amplification of a 439 bp sequence using primer pair OCRAF/OCRAR and confirming them as *A. ochraceus*. In line with the present study, *A. flavus* from wheat flour was identified by using FLA1/FLA2 primer pair with a PCR amplification product of 500 bp [34]. In another study, ITS-based primers were used for the selective identification of black *Aspergilli*. ITS1/NIG primer pair was used for *A. niger* and CAR1/CAR2 primer pair for *A. carbonarius,* and selected primer pairs allowed for the successful differentiation of two species [35]. In line with the present study, *A. flavus*, *A. parasiticus*, *A. niger* and *A. carbonarius* isolated from the animal feed samples marketed in Qatar were identified using species-specific primers [10]. These findings confirm the accuracy and suitability of PCR-based identification for toxigenic fungi. 

### 2.4. Levels of AFs and OTA in Cereals

Human exposure to mycotoxins is mainly due to the ingestion of grains and grain-based products [5]. There are several reports on mycotoxin occurrence in cereal grains and legumes. The levels of mycotoxins depend on the types of cereals and climatic conditions, in addition to many other factors. In Nigeria, Makun et al. [36] detected AFs at 34.1 μg/kg and OTA at 188.2 μg/kg in rice. In the present study, among the tested cereal samples, 7 samples (41%) of wheat and 10 (71%) of rice were positive for AF contamination. However, all the samples (100%) of maize, oats and breakfast cereals were found to be contaminated with AFs (Table 2).

The aflatoxin contamination for different cereals was in the range of nd–3.14 μg/kg for wheat, nd–3.50 μg/kg for rice, 1.93–6.70 μg/kg for maize, 1.8–2.58 μg/kg for oats and 2.23–3.62 μg/kg for breakfast cereals. None of the cereal samples showed aflatoxin levels higher than the EU maximum limit of 4 μg/kg. 

Since maize is intended to be further processed before human consumption, its set limit is 10 μg/kg [37,38]. None of the tested samples showed levels higher than this limit. The European Union maximum levels for cereals excluding maize are 4 μg/kg, while maize that is intended to be further processed before human consumption has levels of 10 μg/kg [37,38]. Although all the tested samples of maize, oats and breakfast cereal samples were positive for AF contamination, the levels were within the EU’s permissible limits. This high percentage of samples showing AF contamination is predominantly related to the occurrence of aflatoxigenic fungi in the maize, oats and mixed-grain cereals (breakfast cereals). *A. flavus* and *A. parasiticus* are commonly detected in maize and oats as compared to rice and wheat [30,39]. The differentiation witnessed in the fungal ecology might be responsible for the high incidence of AFs in maize and its products. Moreover, the origin of the samples is an important factor, as in the present work, the majority of the maize and oat samples originated from South East Asia, where the occurrence of aflatoxigenic strains is reported more frequently due to favorable weather conditions and relatively poor cereals storage [30]. 

In the case of OTA contents, all samples (100%) of wheat, rice and maize were found contaminated with mycotoxins, while none of the oats and breakfast cereals showed the presence of OTA (Table 2). Like AFs, none of the samples among the wheat, rice and maize had OTA contents higher than the EU maximum limit of 4 μg/kg. The contamination of cereal within the EU permissible limits indicates the good quality of the cereals marketed in the Qatari market. These findings are in line with those of AbdulKardar et al. [9], who reported similar levels (1.65–1.95 μg/kg) of OTA in rice samples marketed in Qatar. However, in the same study, they did not detect OTA in any wheat, wheat flour or corn flack samples. The findings are in accordance with the incidence of ochratoxigenic fungi, which were likely less commonly found in the rice and oats samples.

### 2.5. Effects of Cyberlindnera Jadinii 273 Volatiles on Fungal Growth 

**Figure 2:**
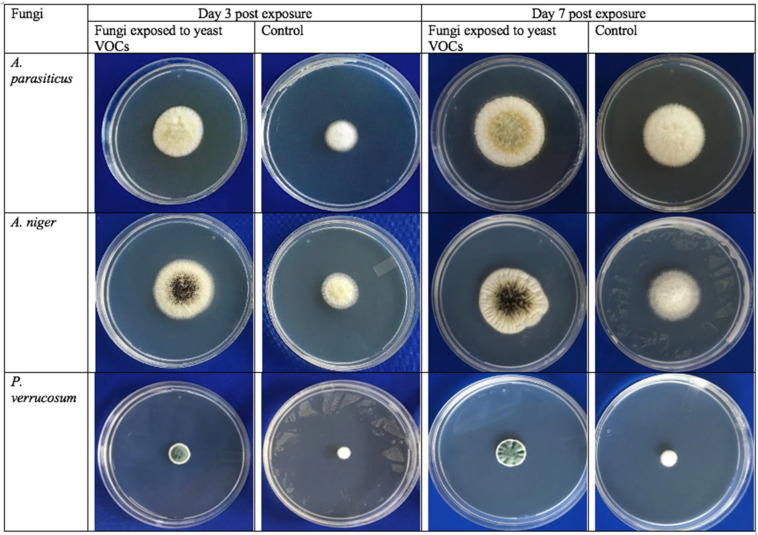
Effect of *Cyberlindnera jadinii* 273 VOCs on the colony sizes and sporulation of the fungal strains isolated from the cereal samples. *A. parasiticus*, *A. niger* and *P. verrucosum* were incubated in the environment of yeast volatiles for 3, 5 and 7 days. Yeast volatiles significantly inhibited fungal growth and sporulation.

**Figure 3:**
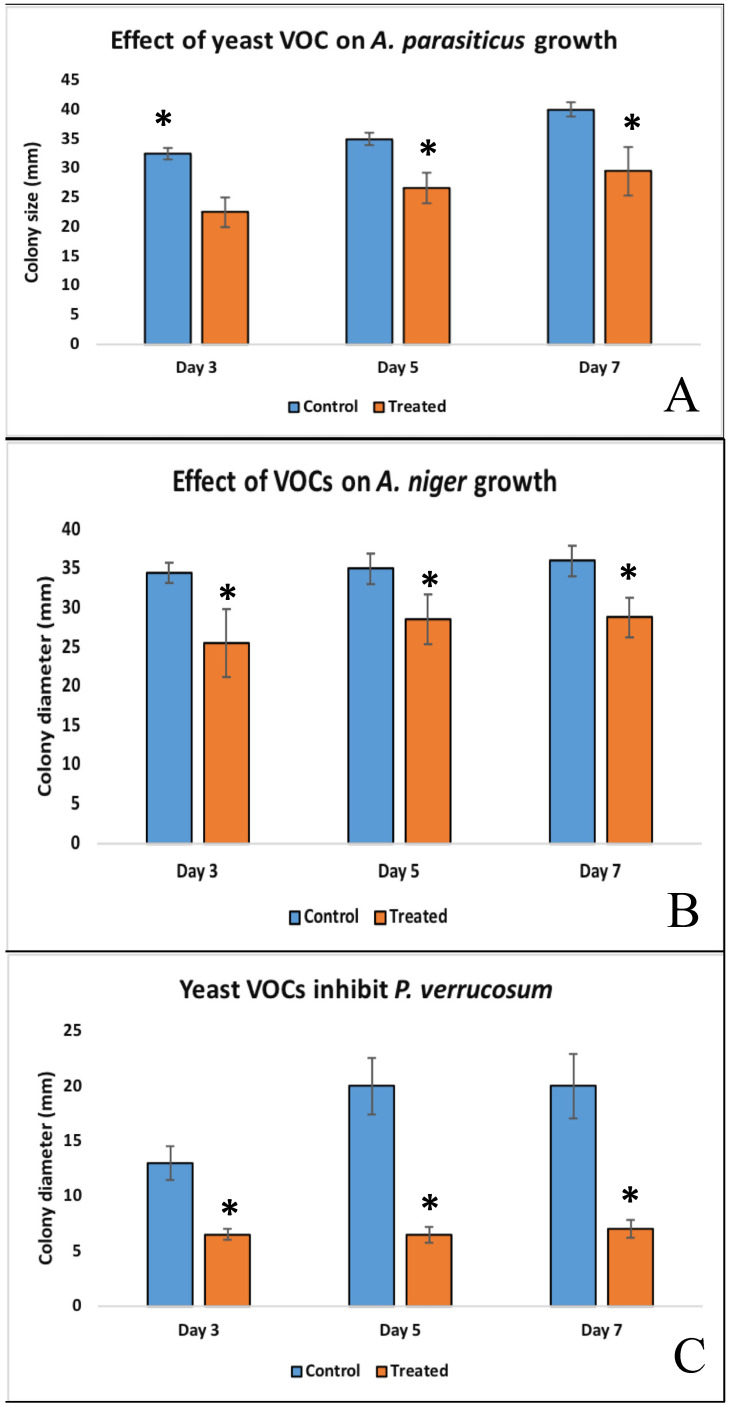
Effect of yeast volatiles on colony diameter of three toxigenic fungi. All three fungal strains *A. parasiticus* (**A**), *A. niger* (**B**) and *P. verricusum* (**C**) were significantly inhibited by *Cyberlindnera jadinii* 273 at all three time points i.e., 3, 5 and 7 days. (*), treated.

In the presence of yeast volatile organic compounds (VOCs), the growth of *A. parasiticus* was significantly inhibited compared to the unexposed control. The effect of yeast volatiles was significant at days 3, 5 and 7 of co-incubation. The diameter of *A. parasiticus* colony exposed to *Cyberlindnera jadinii* 273 VOCs at days 3, 5 and 7 was 22.56 ± 2.51 mm, 26.63 ± 2.67 mm and 29.50 ± 4.21 mm, respectively. These values were significantly lower than the control, whereas at days 3, 5 and 7 the diameters were 32.5 ± 0.9 mm, 35.0 ± 1.0 mm and 40.0 ± 1.2 mm, respectively (Figure 2 and Figure 3A). In line with these findings, diameters of *A. flavus* [23,25] and *A. carbonarius* [40] colonies were significantly reduced on exposure to yeast VOC. The biocontrol activity of the yeast VOCs is attributed to the release of different antifungal compounds, mainly 2-phenylethanol, which is known to inhibit the growth of fungi even at very low concentrations [41]. Another mechanism of inhibition was noted in *Pichia anomala* volatiles, in which the growth of *A. flavus* was inhibited by downregulating the expression of the genes associated with fungal vegetative growth [42].

Similarly, the colony size of *A. niger* was measured at days 3, 5 and 7 of exposure to yeast volatiles and was compared with the control (fungi not exposed to yeast VOCs). As shown in Figure 2 and Figure 3B, yeast volatiles significantly inhibited the growth and sporulation of *A. niger*. At days 3, 5 and 7, the colony size of fungi exposed to yeast VOCs were 25.54 ± 4.32 mm, 28.60 ± 3.2 mm and 29.80 ± 2.5 mm compared to 34.50 ± 1.3 mm, 35.0 mm ± 1.9 and 36.0 ± 2.0 mm for the control, respectively. In compliance with the present study, *B. simplex* volatiles, composed mainly of quinoline and benzenemethanamine, significantly inhibited *A. flavus* and *A. carbonarius* in coffee beans [43]. These findings suggest potential application of *Cyberlindnera jadinii* 273 during storage of food items in commercial as well as domestic settings. 

Finally, *P. verrucosum*, an important ochratoxigenic species for fruits and cereals, was exposed to *Cyberlindnera jadinii* 273 volatile in co-incubation assays. The growth of *P. verrucosum* was monitored at days 3, 5 and 7 in the treated fungi, as shown in Figure 2 and Figure 3C. The colony diameters of the fungi were 6.5 ± 0.5 mm, 6.5 ± 0.75 mm and 7.0 ± 0.81 mm as compared to 13.0 ± 1.5 mm, 20.0 ± 2. 6 mm and 20.0 ± 2.9 mm in the untreated control fungi, respectively. This growth retardation might be associated with the downregulation of genes associated with fungal growth [41,44]. In a similar approach [23], there were significant reductions in the growth and OTA synthesis ability of *P. verrucosum* upon exposure to the VOCs of a low-fermenting yeast, *Lachancea thermotolerans*. At days 3, 5 and 7, the growth of *P. verrucosum* was inhibited at rates of 32%, 37.91% and 43.72%, respectively [23]. These activities are mainly associated with 2-phenylethanol (2-PE), which was a major component of the head-space volatiles of this yeast strain [41]. This assumption can be confirmed from the findings of Tilocca and others [44], where an artificial mixture of commercial 2-PE showed a significant inhibition of toxigenic *A. carbonarius*. 

## 3. Conclusions

In this study, 75 cereal samples (including wheat, rice, maize, oats and breakfast cereals) were tested for the presence of mycotoxins and toxigenic fungi. *Aspergillus* spp. were the most frequent contaminating fungi of the cereals, with the highest isolation frequency 100% in maize samples. The occurrence of key toxigenic fungi, such as *A. flavus*, *A. parasiticus*, *A. ochraceus*, *A. carbonarius* and *A. niger,* was confirmed by their morphological characteristics and molecular profiles. All types of cereals were contaminated with AFs, with significantly higher levels in maize samples. Levels of OTA were negligible in all cereals and were non-significant in wheat, rice and maize samples. Overall, none of the toxins in any of the cereals was higher than the EU’s permissible limits, suggesting no expected threat of mycotoxin exposure to humans. The volatiles of *Cyberlindnera jadinii* (an antagonistic yeast) significantly inhibited the growth and spread of the *A. parasiticus*, *A. niger* and *P. verrucosum* strains. These findings suggest the presence of toxigenic fungi and mycotoxins in marketed cereals and the biocontrol activities of *Cyberlindnera jadinii* against toxigenic fungi, which potentially can replace the application of synthetic fungicides in the agriculture and food industry. 

## 4. Materials and Methods

### 4.1. Material and Supplies

*Cyberlindnera jadinii* 273 was kindly provided by Prof. Quirico Migheli, UNISS, Italy. RIDASCREEN^®^ Ochratoxin A 30/15 and Aflatoxin Total ELISA kits and data reduction software R9996 RIDA^®^ were obtained from R-Biopharm, Dermstadt, Germany. Tecan Sunrise^®^ microplate absorbance reader (Tecan, Grödig, Austria). The fungal isolation media, potato dextrose agar (PDA), Czapek Dox Yeast Extract Agar (CYA), Malt extract agar (MEA), Dichloran Rose Bengal Chloramphenicol Agar (DRBC) and Glycerol 25% nitrate (G25N) were all purchased from Sigma, Dermstadt, Germany. All the buffer solutions were prepared in the lab. 

### 4.2. Sampling

In total, 75 samples of cereals, including wheat grain (n = 17), rice (n = 14), maize (n = 13), oat (n = 14) and breakfast cereals (n = 17) were collected from the local markets in Doha (Qatar). Since toxigenic fungi and mycotoxins have a heterogeneous distribution in food, the contents of bags were well mixed before collecting a representative sample. Each sample of food item was collected from each different lot at three different points and pooled together to generate at least 1 kg of final sample. All the samples were aseptically transported to the research lab at Qatar University and kept at 4 °C prior to analysis. 

### 4.3. Isolation and Morphological Identification of Fungi

After surface disinfection of the grains with 1.5% bleach, they were directly plated on DRBC agar plates. Based on the size of grains, up to 20 grains were aseptically placed on the media. In case of powdered samples, dilution plating method of Pitt and Hocking was adopted [30]. Samples were diluted in sterile distilled water and 100 µL was plated on DRBC media plates. All the plates were incubated at 28 °C for five days. In the case of grains, the fungal infection was calculated in percentage (%) by using following the formula:

$$Grains\;infection\;\left(\%\right)=\frac{No.\;of\;grains\;showing\;at\;least\;a\;single\;fungal\;sp.\;}{Total\;No.\;of\;grains}\times 100$$

For the ground samples (powder), the fungal infection was calculated as CFU/g of the cereal samples [10].

To calculate the isolation frequency (*Fr*) of *Aspergillus* and *Penicillium* in the cereal samples, the following equation was used:

$$\begin{array}{c} Fr\;\left(\%\right)of\;Aspergillus\;or\;Penicillium\\ \;=\frac{No.\;of\;samples\;with\;a\;specie\;of\;genus}{Total\;No.\;of\;samples}\times 100 \end{array}$$

However, relative density (*RD*) of genus *Aspergillus* and *Penicillium* was calculated by the following formula:

$$RD\;\left(\%\right)=\frac{No.\;of\;isolates\;of\;Aspergillus\;or\;Penicillum}{Total\;No.\;of\;Isolates}\times 100$$

All the fungal strains were purified by monosporic isolation using the method described by Balmas et al. [45]. Pure strains were transferred to identification media CYA, MEA and G25N and were incubated at 30 °C, 37 °C and 5 °C for 7 days. Colony morphological characteristics, such as size, color, extent of sporulation, and microscopic features, were all pooled to match the *Aspergillus* and *Penicillium* key [30]. 

### 4.4. Molecular Identification of Fungi Isolated from the Cereal Samples

Fungal DNA was extracted using QIAGEN Plant DNeasy kit as described by Hassan et al. [10]. Briefly, fungal spores from five-day-old pure colonies were suspended in 100 mL of potato dextrose broth (PDB) and incubated in shaking incubator (180 RPM) at 28 °C for 24 hrs. Freshly growing mycelia were obtained by filtration using Whatman No. 1 filter papers. Colonies were ground to powder in liquid nitrogen and DNA extraction protocol was followed as described in QIAGEN manual. Extracted DNA was tested for suitability in PCR reaction using ITS1 (TCC GTA GGT GAA CCT GCG G) ITS4 (TCC TCC GCT TAT TGA TAT GC) primers. For the specific identification of mycotoxigenic species, primer pair FLA1 (GTAGGGTTCCTAGCGAGCC) FLA2 (GGAAAAAGATTGATTTGCGTC) for *A. flavus*, PAR1 (GTCATGGCCGCCGGGGGCGTC), PAR2 (CCTGGAAAAAATGGTTGTTTTGCG) for *A. parasiticus*, ITS1 (TCCGTAGGTGAACCTGCGG) NIG (CCGGAGAGAGGGGACGGC) for *A. niger*, CAR1 (GCATCTCTGCCCCTCGG) CAR2 (GGTTGGAGTTGTCGGCAG) for *A. carbonarius*, OCRAF (CTTTTTCTTTTAGGGGGCACAG), OCRAR (CAACCTGGAAAAATAGTTGGTTG) for *A. ochraceus* and WESTF (CTTCCTTAGGGGTGGCACAG), WESTR (CAACCTGATGAAATAGATTGGTTG) for *A. westerdijkiae* were used. All the PCR mixes and conditions were adopted [10]. Amplified PCR products were analyzed by 1% agarose gel DNA electrophoresis and visualized by UV. 

### 4.5. Monitoring of Aflatoxins (AFs) and Ochratoxin A (OTA) in Cereal Samples

Mycotoxin analysis was carried out using ELISA kit procured from R-Biopharm, Germany. Manufacturer’s guidelines were followed for the extraction of mycotoxins from the cereal matrices. For the extraction of AFs, cereals samples were suspended in methanol and filtered [10]. After dilution, 50 µL of the analyte was used in ELISA wells. On the other hand, cereal samples were first acidified with 1 N HCl and then suspended in dichloromethane (DCM) for the extraction of OTA. Samples were diluted in dihydrogen carbonate buffer before loading in ELISA wells. All the absorbance values were measured at 450 nm and values of the unknown samples were calculated by generating a calibration curve based on the absorbance values of known standard solution provided with the kits. The software RIDA^®^SOFT Win (Art. No. Z9999) was used for these calculations.

### 4.6. Biocontrol of Toxigenic Fungi Using Antagonistic Yeast (Cyberlindnera jadinii 273) Volatiles

The antagonistic yeast (*Cyberlindnera jadinii* 273) was used to produce antifungal volatile organic compounds (VOCs) to inhibit the growth of selected toxigenic fungi isolated from the cereal samples. For this purpose, yeast cells were pre-cultured on yeast extract peptone dextrose broth (YPDB) for 24 hrs. A suspension of 100 µL of yeast cells was plated on YPDA plates and incubated at 28 °C for 24 hrs [40]. For co-incubation assay, the lid of yeast plates was replaced by bottom PDA plates freshly inoculated in the center with 10 µL spores of *A. flavus*, *A. niger* and *P. verrucosum*. After tight sealing to inhibit the evaporation of VOCs and allow the direct exposure of fungal spores to yeast compounds, the plates were incubated at 28 °C. Fungi in the control plates were incubated with YPDA media without inoculated yeast cells. After days 3, 5 and 7 of incubation, colony diameters of treated fungi were measured and compared to the control. Moreover, fungal growth inhibition ration (FGI %) was calculated as given below.

$$Fungal\;growth\;inhibition\;\left(\%\right)=\frac{C-T}{C}\times 100$$

where, *C* is the diameter (mm) of control fungi and *T* is the diameter of treated fungi at different time points. In total, 12 replicates were prepared for each fungus and its respective controls. 

### 4.7. Statistical Analysis

The analysis of variance test (ANOVA) was performed using SPSS software (ver. 23). Different group means were compared either by Duncan’s multiple range (DMR) test or by student *t*-test [46]. Colony-forming units (CFU/g), relative density (RD), isolation frequencies and fungal growth inhibition ratios were calculated by using specific equations given in their respective sections.

## Figures and Tables

**Table 1:** Frequency (Fr) and relative density (RD) of *Aspergillus* and *Penicillium* in the cereal samples.

Item	Fungi Isolated	No. of Isolates	Isolation Frequency (%)	Relative Density (%)
Wheat (n = 17)	*Aspergillus*	25	88.23 ± 13.33 ^a^	67.56 ± 11.73
	*Penicillium*	3	11.76 ± 5.21	8.57 ± 2.27 ^b^
Rice (n = 14)	*Aspergillus*	17	78.57 ± 14.81 ^a^	73.91 ± 16.20
	*Penicillium*	2	14.28 ± 6.67	8.69 ± 0.94 ^b^
Maize (n = 13)	*Aspergillus*	31	100 ± 0.00 ^a^	65.95 ± 8.59
	*Penicillium*	7	30.76 ± 6.53	14.89 ± 3.36 ^a^
Oats (n = 14)	*Aspergillus*	5	14.28 ± 3.25 ^b^	55.56 ± 7.31
	*Penicillium*	0	0	0
Breakfast Cereals (n = 17)	*Aspergillus*	0	0	0
	*Penicillium*	0	0	0

The frequency (Fr) and relative density of the isolated *Aspergillus* and *Penicillium* species were determined in cereal samples by using the formulas given in methodology sections. *Aspergillus* spp. are found in abundance in cereal samples except breakfast cereals. *Penicillium* was the least present in all cereals, with no contamination in oats and breakfast cereals. Values in columns with different superscripts are significantly different from each other at *p* ≤ 0.05.

**Table 2:** Mycotoxin levels in different cereal samples.

Item	Mycotoxin Concentration (μg/kg)			
	Aflatoxins (AFs)		Ochratoxin A (OTA)	
	Range (Min–Max)	Mean ± SD	Range (Min–Max)	Mean ± SD
Wheat (n = 17)	ND *–3.14	2.81 ± 1.21 ^b^	1.91–2.79	2.31 ± 0.43
Rice (n = 14)	ND–3.50	2.23 ± 1.07 ^b^	1.84–2.91	2.41 ± 1.43
Maize (n = 13)	1.93–6.7	3.89 ± 1.32 ^a^	1.57–2.58	2.05 ± 0.89
Oats (n = 14)	1.8–2.58	1.87 ± 0.86 ^b^	0	0
Breakfast Cereals (n = 17)	2.23–3.62	2.59 ± 1.60 ^b^	0	0

The values in the columns with different superscript letters are significantly different from each other at *p* ≤ 0.05; * ND = not detected.

## Data Availability

Data can be provided by the corresponding author on email request.
